# Cinnamic Derivatives as Antitubercular Agents: Characterization by Quantitative Structure–Activity Relationship Studies

**DOI:** 10.3390/molecules25030456

**Published:** 2020-01-21

**Authors:** Cátia Teixeira, Cristina Ventura, José R. B. Gomes, Paula Gomes, Filomena Martins

**Affiliations:** 1 LAQV-REQUIMTE, Departamento de Química e Bioquímica da Faculdade de Ciências da Universidade do Porto, P-4169-007 Porto, Portugal; 2 Instituto Superior de Educação e Ciências, P-1750-142 Lisboa, Portugal; 3 CICECO, Departamento de Química, Universidade de Aveiro, P-3810-193 Aveiro, Portugal; 4 Centro de Química e Bioquímica (CQB), Centro de Química Estrutural (CQE), Faculdade de Ciências da Universidade de Lisboa, P-1749-016 Lisboa, Portugal

**Keywords:** antitubercular agents, cinnamic acids, multi-linear regression analysis, *Mycobacterium tuberculosis*, QSAR model

## Abstract

Tuberculosis, caused by *Mycobacterium tuberculosis* (*Mtb*), remains one of the top ten causes of death worldwide and the main cause of mortality from a single infectious agent. The upsurge of multi- and extensively-drug resistant tuberculosis cases calls for an urgent need to develop new and more effective antitubercular drugs. As the cinnamoyl scaffold is a privileged and important pharmacophore in medicinal chemistry, some studies were conducted to find novel cinnamic acid derivatives (CAD) potentially active against tuberculosis. In this context, we have engaged in the setting up of a quantitative structure–activity relationships (QSAR) strategy to: (i) derive through multiple linear regression analysis a statistically significant model to describe the antitubercular activity of CAD towards wild-type *Mtb*; and (ii) identify the most relevant properties with an impact on the antitubercular behavior of those derivatives. The best-found model involved only geometrical and electronic CAD related properties and was successfully challenged through strict internal and external validation procedures. The physicochemical information encoded by the identified descriptors can be used to propose specific structural modifications to design better CAD antitubercular compounds.

## 1. Introduction

Tuberculosis (TB), caused by *Mycobacterium tuberculosis* (*Mtb*), is one of the most devastating infectious diseases, which currently still has high mortality levels [1]. Despite the availability of treatments, 7 million new cases and 1.5 million deaths are the alarming figures recently reported by the World Health Organization (WHO) for 2018 [1]. The massive resurgence of multi- and extensively-drug resistant TB, together with the high susceptibility of HIV-infected persons to the disease, are current important concerns leading to an urgent demand for new and more effective antitubercular drugs [2,3,4,5]. One of the approaches being used for this purpose is the identification of new therapeutic uses (repurposing) for molecules that were already approved to treat a specific disease or were previously synthesized but were not found to have a clinical application [6]. This is the case of the fluoroquinolones gatifloxacin and moxifloxacin, marketed in 1999 for the treatment of respiratory tract infections, and which are presently the most valuable second-line anti-TB agents according to the WHO guidelines [7].

Cinnamic acid derivatives (CAD) have a century-old history as antitubercular agents [8]. However, this family of compounds was never fully explored for their antimicrobial activity against *Mtb*, and it was not until a decade ago that some studies were conducted to find novel CAD active against tuberculosis [8,9,10,11,12,13,14,15,16]. Noteworthy, trans-cinnamic acid was found to be bacteriostatic at 200 μg/mL against *Mycobacterium smegmatis* [9] and was reported to show synergism with some first-line antitubercular agents [9,10,12]. As the cinnamoyl scaffold is a privileged and important pharmacophore in medicinal chemistry, in recent years CAD have also attracted much attention due to their antitumoral, antioxidative, and antimalarial properties [17,18,19]. In this context, and following our experience in the establishment of biologically relevant quantitative structure–activity relationships (QSAR) [20,21,22,23], we have engaged in the setting up of a QSAR strategy to: (i) derive a statistically significant model to describe the antitubercular activity of CAD towards wild-type (*wt*) *Mtb*; and (ii) identify the most relevant properties that have a substantial effect on the antitubercular activity of those derivatives.

QSAR analysis is usually based on the assumption that compounds with similar structures are expected to exhibit similar properties and, therefore, changes in chemical structure are likely to be accompanied by proportional changes in biological activity. Although it is now widely known that this congeneric principle is not as universal as initially thought [24,25,26], it is still the basis behind many computational QSAR studies, from the time when Hansch established the very first QSAR model to predict chemical solubility up to these days [27]. Due to the current explosive growth of experimental data, mainly originated from high throughput screening campaigns, several QSAR methodologies have been called up to establish models involving large and complex data sets [28,29,30].

Differences among the various QSAR approaches depend mostly on the descriptors used to characterize the molecules and the methods used to establish relationships between input descriptor values and biological activities. The choice of a particular method depends mainly on the nature of the problem being addressed and on the final purpose of the analysis [22,23]. Linear methods are usually used if the main objective is to rationalize and/or interpret a given biological behavior, while nonlinear methods are more commonly employed if the main purpose is to accurately predict a property. However, non-linear methods are prone to overfitting, which occurs when the number of descriptors (ranging from hundreds to thousands) is much greater than the number of samples in the dataset (less than a hundred compounds is common). In this context, as we were handling a small modeling dataset, we chose a multiple linear regression (MLR) analysis, which is one of the most used linear methods to build up QSAR models and has been very profusely and successfully applied in the field of Medicinal Chemistry [31]. Additionally, MLR has the advantage of being easily interpretable, allowing a direct link between a given biological response and the set of molecular features, encoded by the descriptors, which are responsible for that response. In this work, we depict the details of the construction and validation of an MLR-based model to describe the antitubercular activity of a set of CAD and the analysis of the model’s descriptors.

## 2. Results and Discussion

A dataset of 54 CAD with known MIC values for the *Mtb* H37Rv strain was retrieved from ChEMBL [32]. Two different research groups performed the in vitro experiments [10,11,14,16]. However, a QSAR dataset should include biological activity values for all compounds, preferably measured using the same experimental methodology. As this was not the case, and as the quality of the input data has a large influence in the QSAR model quality, the set with the higher number of compounds was selected in this study [11,16]. Thus, the final data set comprised 29 compounds covering a wide range of MIC values from 0.26 to 1560.59 μM. A pool of 33 molecular descriptors (energetic, geometrical, structural, physicochemical, electronic) was generated using MMPro+ or ChemDraw for cLogP (see experimental section for details). The data set was then split into a training set (22 compounds) and an independent test set (7 compounds) as indicated in Table 1. Compounds were selected in such a way that the chemical domain in the two sets was not too dissimilar and that both the training and the test sets spanned, separately, the entire descriptor space occupied by both sets (Supplementary Materials Table S1). The training set was used to derive the model, whereas the test set was used to evaluate the predictive ability of the generated model.

To establish a relationship between the MIC value and the molecular characteristics of the training set compounds, we derived standard MLR-based QSAR models, testing all combinations of the 33 descriptors and retaining or disregarding descriptors according to rigorous statistical criteria. The intercorrelation matrix among descriptors was always checked; descriptors were considered not intercorrelated, and therefore non-redundant, if *r*^2^ between any two descriptors was below 0.5 and *R*^2^ of one against all others was below 0.8 [21,22,33]. Suspicious points were initially spotted by inspection of a plot of Y_calc_ vs. Y_exp_ and then confirmed as outliers according to two criteria: the conventional measure |Y_calc_ − Y_exp_| > 2 SD, where SD stands for standard deviation of the fit, and a more refined measure known as the Cook’s distance (see experimental section for details) [22]. The identified outliers were compounds **10**, **17,** and **28**, as seen in Table 1. The occurrence of outliers can happen for many reasons, such as: i) an error in the reported MIC value or in one or several derived descriptors’ values; ii) a mechanism of action different from that of the majority of the data set points; or iii) a non-representative sampling design, among others. Still, no plausible explanation could be assigned for the outlier behavior of compounds **10**, **17,** and **28**. The best model, found by a forward stepwise procedure, upon removal of these three compounds from the training set, is shown in Table 2.

The model’s robustness (evaluated with training set compounds) was duly assessed, and the best found model fulfilled all the recommended criteria for internal validation (Table 3) such as a determination coefficient (*R*^2^) higher than 0.6, a leave-one-out (LOO) cross validation correlation coefficient (*Q*^2^_LOO_) higher than 0.6, the *F*-test value (*F* = 35) significant at 99% with its corresponding tabulated value, a small value for the standard deviation of the fit (*SD* = 0.357), and a significance level (SL) of each adjusted parameter higher than 95% [21,29,34,35]. In order to remove any possibility of attributing the quality of the statistics of these models to a chance correlation between the response variable and the descriptors, a Y-randomization test was performed on the developed QSAR model (Supplementary Materials Table S2). We observed a significant decrease in the quality of the randomized models when compared to the original non-randomized one, and therefore it seemed there was no chance correlation, as corroborated by the value of ^c^*R*^2^_p_, quite above the 0.5 threshold value [36]. Chance correlation was also assessed by applying the Q under influence of K (QUIK) rule, a technique that measures the total correlation of a set of variables and that allows the rejection of models with high predictor collinearity, as proposed by Todeschini [37]. Thus, according to the QUIK rule, our model was not due to chance correlation, as the *xy* correlation was higher than the *x* correlation (Supplementary Materials Table S2). Finally, the absence of intercorrelation between descriptors in the best model (Supplementary Materials Table S3) also indicated that the quality of the statistics was not due to collinearity among descriptors.

**Figure 1:**
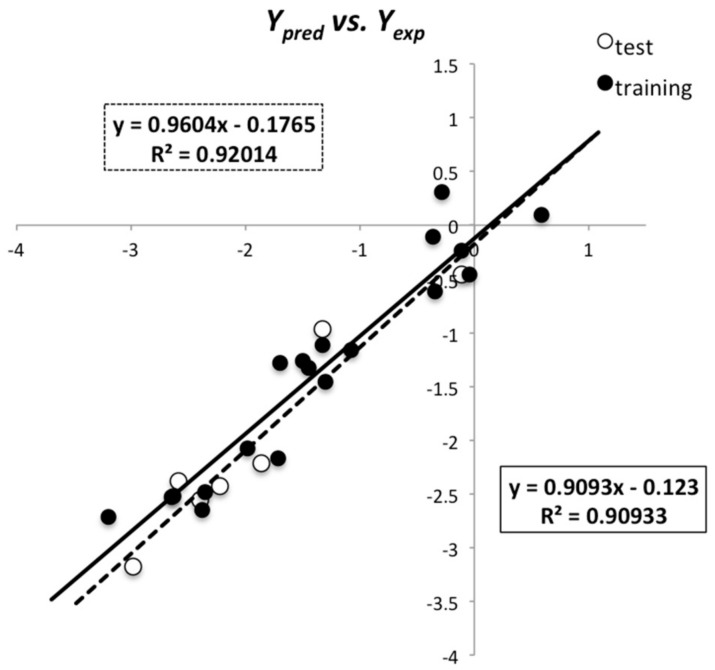
Log(1/MIC)_pred_ vs. log(1/MIC)_exp_ according to the best built QSAR model.

Internal validation methods, as the ones mentioned above, are very useful to assess whether a model is stable and robust, and whether overfitting occurs. However, it is more and more commonly accepted that the predictive power of a QSAR model should be evaluated by verifying if the model is able to predict the behavior of chemicals not used on the training set. For that purpose, the predictive ability of the best-found model was analyzed using the test set. The external predictivity was confirmed as the established QSAR model fulfilled all the following recommended “classic” criteria for external validation (Table 3): *Q*^2^_ext_ > 0.5, *R*^2^ > 0.6; (*R*^2^ – *R*_0_^2^)/*R*^2^ < 0.1; 0.85 < *m* < 1.15, where *R*_0_^2^ is the test set’s regression determination coefficient that goes through the origin, and *m* is the slope of the regression between the predicted and the experimental values. The parameter *r*_m_^2^, proposed by Roy and Paul [29,38,39], was also used to assess external validation of the model (Table 3). This stricter parameter penalizes a model for large differences between predicted and experimental values of the test set compounds not accounted for by *Q*^2^_ext_, being an indicator of good external predictivity if greater than 0.65. External validation was also performed by using the very demanding concordance correlation coefficient (CCC) [40,41]. This coefficient measures both accuracy (how far the regression line deviates from the concordance line) and precision (how far the observations are from the fitting line) between experimental and predicted values (Table 3), and a minimum value of 0.85 is required as an indicator of good predictive ability. Finally, a scatter plot of predicted vs. experimental values was also obtained, as recent studies have recommended the visual inspection of these plots as important complementary indicators of model predictivity [22,41]. The scatter plot for the best-found model is represented in Figure 1 showing that no systematic deviations from the ideal line were observed.

A close analysis of Table 2 reveals that the activity of the studied CAD against *wt Mtb* H37Rv strain did not depend on their energetic, steric, or physicochemical features. Descriptors belonging to these classes were found not to contribute to model log (1/MIC) values. Additionally, the lipophilicity, as measured by cLogP, did not seem significant to explain the antitubercular activity of this family of compounds. On the other hand, geometrical and electronic properties came out as very effective in explaining the biological activity of these derivatives. Indeed, the best-found model included two geometrical descriptors (angles ***a*_1_** and ***a*****_3_** as depicted in Table 2), which both favored activity, suggesting that the sp^2^ hybridization for these sets of atoms is preferred over the sp^3^. The model also comprises two properties related to the ability of permeation through membranes, polar surface area (PSA) and Hanse polarity parameter (HansPol). This last parameter represents the energy from dipolar intermolecular interactions and contributes negatively to the antitubercular activity of the CAD. Conversely, PSA, which corresponds to the surface sum over all polar atoms (primarily oxygen and nitrogen, including their attached hydrogens), contributes to enhanced activity with an average of 71.3 Å^2^ for training set and 73.2 Å^2^ for test set compounds, thus indicating that compounds that are good at permeating membranes are preferred. However, both geometrical descriptors have a relatively higher impact than the two electronic descriptors in the activity of these compounds. Although the cinnamic skeleton has been considered an interesting scaffold for the development of novel antimicrobials, little is known about its mechanism of antimicrobial action. Therefore, no clear relation between a possible mode of action of this family of compounds and the model’s descriptors can be made. Still, the results clearly suggest that both penetration through cell membrane and adequate geometrical properties to bind to their target are crucial for the biological activity of CAD.

**Figure 2:**
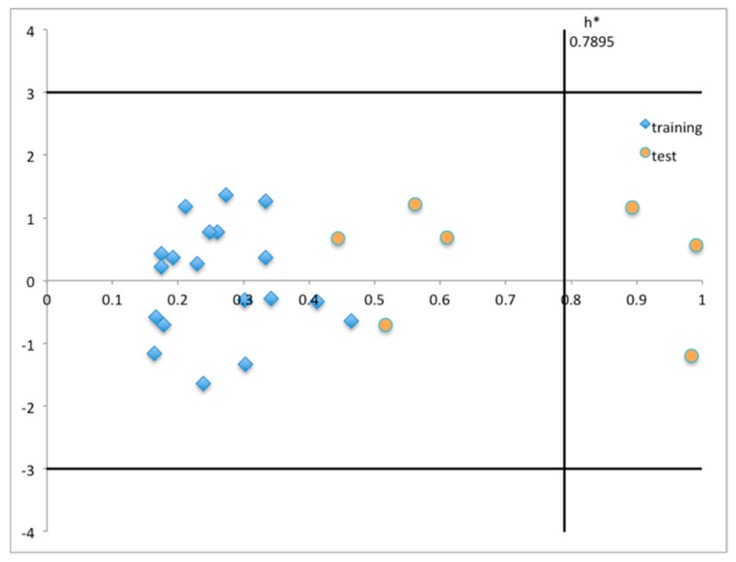
Williams plot for the built model representing the leverage values for the training and test set compounds.

An additional important aspect to take into consideration is the applicability domain (AD) of the built MLR model, which is crucial to ensure that its predictions are reliable. Thus, to assess the AD for the QSAR model obtained, two different methods were considered: i) the leverage approach (Figure 2) [42], and ii) the range of the individual descriptors (Supplementary Materials Table S1). The leverage value, *h*, provides a measure of the distance of a molecule from the training set’s centroid. A “cautionary leverage”, h*, is usually set to 3*p*/*N*, where *N* is the number of molecules in the training set and *p* the number of model descriptors plus one [42]. Thus, plotting the standardized residuals, *SR*, as a function of the leverage values (Williams plot) allows for a graphical assessment of the AD, enabling the detection of influential points, i.e., compounds structurally distant from training set compounds (*h* > h*) and of response outliers (*SR* > ± 3 *SR* units). In a Williams plot, the AD is defined by the squared area between ± 3 SR and the threshold h* value. By analyzing Figure 2, we can observe that the built MLR model performed well in terms of AD. In fact, training and test set compounds lie within ± 3 *SR* units, indicating the absence of any outlier. Additionally, there are also no significant influential points in the training set since the leverage values of all compounds are smaller than the cut-off value h*. Three compounds from the test set (**12**, **23,** and **24**) had *h* values higher than the warning value h*, thus falling somewhat outside the model AD. Still, the model accurately predicted these compounds (*SR* within ± 1.2 units), being such points being called “good influential points” [43].

In summary, a set of 29 CAD was investigated to relate their antitubercular activity values to their molecular structure. Using MLR analysis, a stable and predictive QSAR model with good statistical results was developed. The main descriptors involved in the model were related to geometrical and electronic CAD properties. However, more in-depth studies regarding the mechanism of action of the antitubercular activity of CAD should be performed in order to further explore the relationship between QSAR model descriptors and the physicochemical properties of the surface of bacterial cells. Still, the physicochemical meaning of the descriptors of the proposed model will be helpful for rational structural modifications on this class of compounds in order to design better antitubercular agents.

## 3. Materials and Methods

### 3.1. Data Set Preparation and Descriptors Calculation

The data set consisted of 29 cinnamic acid derivatives retrieved from ChEMBL [32] with known MIC values for *Mtb* H37Rv strain (Supplementary Materials Table S4) [11,16]. MIC values were converted to the pMIC scale (–log MIC). MarvinSketch was used for molecules’ construction and the dominant protonation states of molecules were calculated using the Major Microspecies Plugin, MarvinSketch 16.2.1, ChemAxon [44, 45, 46, 47].

A pool of 33 molecular descriptors (Supplementary Materials Table S4) was generated using Molecular Modeling Pro Plus software [44]. Each compound was first submitted to a molecular structure optimization by MM2, a molecular mechanics method incorporated in the software. ChemDraw was used to calculate cLogP values [48].

To determine a relationship between the molecular descriptors of the selected compounds and their respective biological activity, we performed a standard MLR of the type

Y = AX + ζ,(1)

where ζ is an *n* × 1 residuals vector whose elements are assumed to be independent normal random variables with mean zero and known variance σ^2^, X is a known *n* × *k* matrix of molecular descriptors, A is a *k* × 1 vector of adjusted parameters, and Y is an *n* × 1 vector of the response variable related, in this case, to the biological activity. For this purpose, we used the Microsoft Excel Data Analysis add-in and several statistical validation tests to ensure the trustworthiness of the analyses.

### 3.2. Outlier Search

The decision to consider a given point as an outlier was made according to two criteria: Cook’s distance and the more conventional measure |Y_calc_ − Y_exp_| > 2 SD, where SD stands for standard deviation of the fit.

Cook’s distance, *D*i, is a measure of the influence of a suspicious point (outlier) in the results of a certain regression and is given by [45,46]

(2) $$D_{i}=\;\frac{\sum_{i}{\left(\hat{Y\;}-\;{\hat{Y}}_{i}\right)}^{2}}{k\sigma^{2}}\;,$$

where $\hat{Y}$ and ${\hat{Y}}_{i}$ are the *n* × 1 vectors of the predicted observations for the entire data set and for the data set without the ith observation, respectively, and *k* is the number of parameters adjusted by the linear model with a variance σ^2^. The specific criterion used to exclude a supposed outlier was *D_i_* > 4/(*n* – *k* – 1), where *n* is the number of experimental points.

### 3.3. Internal Validation

The data set was divided into training (22 compounds) and test (7 compounds) sets with similar degrees of variability. In order to make an internal validation of the data, we applied the leave-one-out (LOO) approach to the training set [22,34,35] as follows:

(3) $$Q^{2}=1-\;\frac{\sum_{i=1}^{training}{\left(\;y_{i}-\;{\hat{y}}_{i}\right)}^{2}}{\sum_{i=1}^{training}{\left(\;y_{i}-\;{\bar{y}}_{i}\right)}^{2}},$$

where *y*_i_, ${\hat{y}}_{i}$ and ${\bar{y}}_{i}$ are the measured, predicted, and averaged (over the whole data set) values of the dependent variable, respectively, and *Q*^2^ is a cross-validated correlation coefficient.

We also considered traditional statistical criteria such as the determination coefficient, *R*^2^, the standard deviation, SD, the *F* statistic, and the significance level, SL, of each adjusted parameter (parameters were kept if SL > 95%) and tested the intercorrelations among all descriptors included in each regression.

### 3.4. External Validation

The test set was used for external validation, and the predictive ability of the model was assessed by an external *Q*^2^_ext_ parameter defined as [22,34,35]:

(4) $$Q_{ext}^{2}=1-\;\frac{\sum_{i=1}^{test}{\left(\;y_{i}-\;{\hat{y}}_{i}\right)}^{2}}{\sum_{i=1}^{test}{\left(\;y_{i}-\;{\bar{y}}_{training}\right)}^{2}},$$

where *y*_i_ and ${\hat{y}}_{i}$ are the experimental and predicted (over the test set) values, respectively, and ${\bar{y}}_{training}$ is the averaged value of the dependent variable for the training set.

To further assess the predictive capability of the established QSAR model, we also computed three measures of fit, namely the average error (*AE*), the absolute average error (*AAE*), and the root-mean square error (*RMSE*). Additionally, we determined Roy’s parameters [38,39,40,41], *r*^2^_m_ and $\bar{r_{m}^{2}}$ and the concordance correlation coefficient (*CCC*) [40,41]. The former two criteria were calculated according to the following formula:

(5) $$r_{m}^{2}={\;R}^{2}\;(1-\;\sqrt{R^{2}-{\;R}_{0}^{2}},\;\bar{r_{m}^{2}}=\;\frac{\left(r_{m}^{2}+{r\prime }_{m}^{2}\right)}{2},$$

where $R^{2}$ and $R_{0}^{2}$ are, respectively, the determination coefficients of the regression function, calculated using the experimental and the predicted data of the prediction set, forcing the regression to pass, respectively, through the origin of the axis ($R_{0}^{2}$) or not ($R^{2}$). $r_{m}^{2}$ is calculated using the experimental values on the ordinate axis, and $r\prime_{m}^{2}$ using them on the abscissa. The latter criterion *CCC* is obtained by

(6) $$\mathrm{CCC}=\;\frac{2\;\sum_{i=1}^{n_{\mathrm{test}}}\left(Y_{i}-\bar{Y}\right)\left({\hat{Y}}_{i}-\bar{\hat{Y}}\right)}{\sum_{i=1}^{n_{\mathrm{test}}}{\left(Y_{i}\;-\bar{Y}\right)}^{2}+\;\sum_{i=1}^{n_{\mathrm{test}}}{\left({\hat{Y}}_{i}-\bar{\hat{Y}}\right)}^{2}+{\;n}_{\mathrm{test}\;}{\left({\hat{Y}}_{i}-\bar{\hat{Y}}\right)}^{2}\;}\;,$$

where $Y_{i}$ and ${\hat{Y}}_{i}$ stand for the abscissa and ordinate values of the plot of experimental vs. predicted values (or, similarly the opposite, which causes no difference), *n* is the number of compounds, and $\bar{Y}$ and $\bar{\hat{Y}}$ correspond to the averages of experimental and predicted values, respectively.

## Figures and Tables

**Table 1:** Structure of the cinnamic acid derivatives (CAD) used to derive the multiple linear regression (MLR)-based model. Compounds marked with an asterisk belong to the test set.

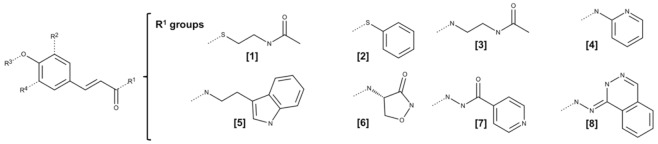											
**Cpd**	**R^1^**	**R^2^**	**R^3^**	**R^4^**	**MIC H_37_Rv** **(μM)^2^**	**Cpd**	**R^1^**	**R^2^**	**R^3^**	**R^4^**	**MIC H_37_Rv** **(μM)^2^**
**1**	[1]	H	farnesyl	H	1.28	*16	[5]	H	isopentenyl	H	168.23
**2**	[1]	H	isopentenyl	H	95.97	**17^1^**	[5]	OCH_3_	H	H	23.78
**3**	[1]	H	methyl	H	225.52	*18	[6]	H	methyl	H	950.00
*4	[1]	OCH_3_	H	OCH_3_	384.16	**19**	[7]	H	isopentenyl	H	2.30
**5**	[1]	OCH_3_	H	H	423.21	**20**	[7]	H	CF_3_	H	1.10
**6**	[1]	H	H	H	237.44	**21**	[7]	H	CF_3_CH_2_	H	2.20
**7**	[2]	OCH_3_	H	H	27.94	**22**	[7]	H	geranyl	H	1.90
**8**	[2]	H	H	H	31.21	*23	[7]	H	ethyl	H	1.30
**9**	[3]	H	geranyl	H	0.26	*24	[8]	H	isopentenyl	H	21.00
**10^1^**	[3]	H	isopentenyl	H	199.11	**25**	[8]	H	CF_3_CH_2_	H	20.00
**11**	[4]	H	isopentenyl	H	51.88	**26**	[8]	H	ethyl	H	12.00
*12	[4]	H	methyl	H	247.75	**27**	[8]	H	CF_3_	H	21.00
**13**	[4]	OCH_3_	methyl	H	439.65	**28^1^**	[8]	H	geranyl	H	72.00
**14**	[5]	H	methyl	H	1560.59	**29**	[8]	H	methyl	H	50.00
*15	[5]	H	geranyl	H	72.30						

^1^ Compound identified as outlier. ^2^ MIC values were retrieved from references [11,16]. * Test set compounds.

**Table 2:** Best model found by MLR analysis: pMIC = a_0_ + a_1_**a3** + a_2_**a1** + a_3_**PSA** + a_4_**HansPol**. Molecular descriptors are in bold, and values in parenthesis correspond to the significance level of each adjusted parameter. All descriptors were normalized.

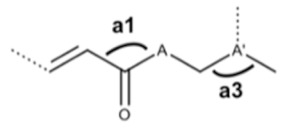				
a_0_ ± s(a_0_) (SL)	a_1_a_3_^1^ ± s(a_1_) (SL)	a_2_a_1_^1^ ± s(a_2_) (SL)	a_3_PSA^2^ ± s(a_3_) (SL)	a_4_HansPol^3^ ± s(a_4_) (SL)
−5.061 ± 0.442 (100.00%)	2.899 ± 0.332 (100.00%)	2.082 ± 0.268 (99.99%)	1.673 ± 0.311 (99.99%)	−1.800 ± 0.354 (99.98%)

^1^**a1** and **a3** correspond to the angles represented in picture of Table 2. ^2^ **PSA**: polar surface area. ^3^ **HansPol**: Hansen polarity.

**Table 3:** Summary of statistical results for the best-found quantitative structure–activity relationships (QSAR) model.

Set	*N* ^1^	*SD* ^2^	*R* ^2 3^	*F* ^4^	*R* ^2^_0_^5^	*AE* ^6^	*AAE* ^7^	*RMSE* ^8^	*Q* ^2 9^	${{\bar{r}}_{m}^{2}}^{10}$	Δ*r*^2^_m_^11^	*CCC* ^12^
Training	19	0.357	0.909	35	-	-	-	-	0.930	-	-	-
Test	7	0.297	0.920	58	0.913	0.100	0.260	0.294	0.933	0.879	0.070	0.953

^1^ Number of compounds. ^2^ Standard deviation of fit. ^3^ Determination coefficient. ^4^ The F statistics.^5^ Determination coefficient of regression through the origin. ^6^ Average error. ^7^ Absolute average error. ^8^ Root-mean square error. ^9^ Cross-validation correlation coefficient. ^10^ Average value between observed vs. predicted and predicted vs. observed Roy’s parameter, *r*^2^_m_, for the test set. ^11^ Absolute difference between observed vs. predicted and predicted vs. observed Roy’s parameter, *r*^2^_m_, for the test set. ^12^ Concordance correlation coefficient.

## Supplementary Materials

The following are available online at https://www.mdpi.com/1420-3049/25/3/456/s1, Table S1: Range of variability of descriptors for the training and test sets, Table S2: Results of the Y-randomization (30 shuffles) and the QUIK rule for the best model, Table S3: Intercorrelation matrix between any two descriptors and between one descriptor and a linear combination of all other descriptors, Table S4: Dataset compounds in SMILES format and respective MIC values (μM) and values of descriptors (values not normalized).
