# Supplementary material for: Cinnamic Derivatives as Antitubercular Agents: Characterization by Quantitative Structure–Activity Relationship Studies
Source: Molecules. 2020 Jan 21;25(3):456. doi: 10.3390/molecules25030456 (PMC7037561; doi:10.3390/molecules25030456)
Supplement: Supplementary file 1 [file molecules-25-00456-s001.zip › Table S1, S2, S3.docx]

Article

Cinnamic Derivatives as Antitubercular Agents: Characterization by Quantitative Structure-Activity Relationship Studies

Cátia Teixeira ^1,^*, Cristina Ventura ^2^, José R. B. Gomes ^3^, Paula Gomes ^1^ and Filomena Martins ^4,^*

^1^ LAQV-REQUIMTE, Departamento de Química e Bioquímica da Faculdade de Ciências da Universidade do Porto, P-4169-007 Porto, Portugal

^2^ Instituto Superior de Educação e Ciências, P-1750-142 Lisboa, Portugal

^3^ CICECO, Departamento de Química, Universidade de Aveiro, P-3810-193 Aveiro, Portugal

^4^ Centro de Química e Bioquímica (CQB), Centro de Química Estrutural (CQE), Faculdade de Ciências da Universidade de Lisboa, P-1749-016 Lisboa, Portugal

***** Correspondence: catia.teixeira@fc.up.pt; feleitao@fc.ul.pt

**Table S1.** Range of variability of descriptors for the training and test sets.

| **Sets** | **Max or min values for descriptors**  **in set** | **pMIC** | ***a3*** | ***a1*** | ***Polar Surface area*** | ***Hanse Polarity parameter*** |
| --- | --- | --- | --- | --- | --- | --- |
| **Training** | max | 6.5849 | 121.721 | 120.818 | 86.64 | 7.7686 |
|  | min | 2.8067 | 109.802 | 112.361 | 40.46 | 2.9472 |
| **Test** | max | 6.5229 | 121.721 | 120.760 | 91.18 | 8.1976 |
|  | min | 3.0223 | 107.325 | 112.127 | 54.38 | 3.0740 |

**Table S2.** Results of the Y-randomization (30 shuffles) and the QUIK rule for the best model.

|  | $R^{2}$ | $R_{r}^{2}$ | $cR_{p}^{2}$ |
| --- | --- | --- | --- |
| Y-randomization | 0.909 | 0.139 | 0.837 |
| QUIK rule | K_xy_ = 35.17 > K_x_ = 30.26 | | |

**Table S3.** Intercorrelation matrix between any two descriptors and between one descriptor and a linear combination of all other descriptors. The outliers were not considered to compute these matrix values.

| Descriptors | a_3_ | a_1_ | PSA | HansPol | Linear combination with all other 3 descriptors |
| --- | --- | --- | --- | --- | --- |
| a3 |  | 0.350 | 0.035 | 0.363 | 0.363 |
| a1 |  |  | 0.022 | 0.004 | 0.353 |
| PSA |  |  |  | 0.065 | 0.109 |
| HansPol |  |  |  |  | 0.080 |
